# Supplementary material for: A qualitative process evaluation of group problem management plus for distressed Syrian refugees in Türkiye
Source: Glob Ment Health (Camb). 2025 Jul 17;12:e87. doi: 10.1017/gmh.2025.10035 (PMC12345057; doi:10.1017/gmh.2025.10035)
Supplement: Coşkun-Toker et al. supplementary material [file S2054425125100356sup001.docx]

**Supplementary Material**

**S1: Background on the gPM+ RCT**

This qualitative process evaluation was conducted in the final phase of a large research study within the STRENGTHS project, and was preceded by an RCT evaluating the effectiveness of the gPM+ intervention among Syrian refugees in Türkiye. Between August 2019 and September 2020, participants were recruited through the Refugees and Asylum Seekers Assistance and Solidarity Association (RASASA), a non-governmental organization (NGO) in Sultanbeyli, İstanbul. 368 eligible participants were randomly assigned to gPM+ or enhanced care as usual. The inclusion criteria were being older than 18 years old, having temporary protection status, being an Arabic speaker and showing elevated levels of psychological stress (measured by the General Health Questionnaire-12) and impaired functioning (measured by WHO Disability Assessment Schedule).

Non-professional Syrian or other Arabic-speaking peer facilitators and co-facilitators (7 female, 3 male) delivered the sessions to groups of eight to ten participants. Based on the recommendations on cultural adaptation process, groups were seperated by gender and the gender of the facilitators was matched by the group. Facilitators were selected if they have completed a minimum of 12 years of education. Most of them were university students at different departments, such as engineeering or social sciences, and had no previous experience in delivering a pscyhological support. They received 8-days classroom training by two PM+ master trainers, completed two practice groups under supervision and participated weekly group supervisions. gPM+ participants received five weekly group sessions at RASASA during the weekend to accommodate their childcare and work commitments. A PM+ supervisor attended ten percent of the sessions to assess treatment delivery. During these sessions and supervisions, none of the facilitators' performances were evaluated as inadequate.

**S2: Topic Guide**

**gPM+ participants**

A) Overall impressions:

- Can you describe your experience of the Group PM+ intervention?

- Explore positive / negative views through probes.

B) Rapport with Group PM+ facilitators:

- Can you describe how you found working with your PM+ facilitator?

- Explore positive / negative views through probes.

- How did your family view your relationship with your Group PM+ facilitators? - Explore positive / negative views through probes.

C) Intervention adherence:

- Can you describe how easy or difficult you found attending weekly group sessions for five weeks? - Explore barriers and facilitators to attendance.

- Can you describe how you found implementing the skills the Group PM+ facilitators taught to you in your everyday routine?

- Explore barriers and facilitators to skills development.

**gPM+ participants’ family members**

- 1. A) Overall impressions:
  2. - Can you describe your experience of the Group PM+ intervention? Or how your family member/friend experienced Group PM+?
  3. - Explore positive / negative views through probes.
  4. B) Rapport with Group PM+ facilitators:
  5. - Can you describe how you found the relationship between your family member/person close to you and the Group PM+ facilitators?
  6. - Explore positive / negative views through probes. - Explore positive / negative views through probes.
  7. C) Intervention adherence:
  8. - Can you describe how easy or difficult it was to attend weekly group sessions for five weeks? - Explore barriers and facilitators to attendance.
  9. - Can you describe how your family member/person close to you found implementing the skills taught by the Group PM+ facilitator in everyday routine? - Explore barriers and facilitators to skills development.

**gPM+ facilitators**

- 1. A) Overall impressions:
  2. - Can you describe your experience of the Group PM+ intervention.
  3. - Explore positive / negative views through probes.
  4. B) Rapport with clients:
  5. - Can you describe your rapport with clients you have supported?
  6. - Explore positive / negative views through probes.
  7. - Can you describe your rapport with families of clients you have supported? - Explore positive / negative views through probes
  8. C) Intervention adherence:
  9. - Based upon your experience, what would help people stay motivated to complete the Group PM+ program?
  10. - To what extent do you feel your clients implemented the skills you taught them through the program?
  11. - Explore barriers and facilitators to skills development.

**Key informants (i.e., a project coordinator, program manager, psychologist, and social worker)**

A) Existing scope of work of primary healthcare facilities:

- Considering existing primary healthcare clinics’ scope of work, do you view this additional intervention as something that can be delivered within the primary healthcare structures?

- Explore barriers and facilitators.

B) Integrating non-specialist facilitators into the PHC structures:

- How do you view the role of non-specialist facilitators within primary healthcare?

- Explore barriers and facilitators to integrating non-specialits facilitators into primary healthcare.

C) Integration of Group PM+:

- What do you think of the Group PM+ program in terms of ensuring high coverage of psychological problems?

- Explore barriers and facilitators

- Into what other programs (other than PHC) can Group PM+ be integrated?

- Explore other available health or social programs/activities available into which this Group PM+ can be integrated

**S3: Analytical coding framework**

| **Theme** | **Code** | **Subcode** | | **Description** | **An illustrative quote from the interviews** |
| --- | --- | --- | --- | --- | --- |
| **1. Views on gPM+ (from participants and family)** | | | | | |
|  | **1.1 Acceptability of gPM+** | | | | |
|  |  | **1.1.1 The content of gPM+** | | Participants mention whether they found the content of gPM+ acceptable | "*I also learned too many things like how to care about my family members and be happy with them and advise them and hear from them.*" -P5 |
|  |  | **1.1.2 The demand for gPM+** | | Participants mention whether they believe they need for or demand gPM+ | "*I did not feel that as I was not in need of such kind of program so I did not feel anything new was added to my life.*" -P4 |
|  |  | **1.1.3 The views of the family members** | | Participants decribe how their family members view their participation in gPM+ | "*My family also supported me, and they noticed that I was very happy to attend this program they told me to join such programs*" -P5 |
|  | **1.2 Implementation of the skills and strategies** | | | | |
|  |  | **1.2.1 Facilitators** | | Participants mention the facilitating factors that would enable them to implement the skills and strategies they learned within the program | "*My family was very supportive and they told me to continue the program as they are seeing some improvement.*" -P3 |
|  |  | **1.2.2 Barriers** | | Participants mention the barriers that would make it challenging for them to implement or prevent them to implement the skills and strategies they learned | "*Yes in some situations I try to apply those skills but I wish the sessions continued so I can practice these skills more with the supervisor..*" -P3 |
|  | **1.3 Views on the group format** | | | | |
|  |  | **1.3.1 Benefits** | | Participants mention the benefits of participating in a group intervention | "*As she was telling me that she was enjoying the program and she was happy about it as she was seeing people and talking to them."* -R3 |
|  |  | **1.3.2 Challenges** | | Participants mention the difficulties of being involved in a group intervention with other group members | "*The people in my session were always asking too many questions that bothered me.*" -P6 |
|  | **1.4 Views on the helpers** | | | | |
|  |  | **1.4.1 Management of the group** | | Participants mention the group management skills of the helpers | "*The program was managed well in all aspects.*" -P1 |
|  |  | **1.4.2 Culture and language** | | Participants mention the cultural competency and the language use of the helpers | "*The providers were Arabic-speaking people so I had no problem at all.*" -P3 |
|  |  | **1.4.3 Competence** | | Participants mention the general competency of the helpers in terms of delivering the sessions | "*The provider was good as well and was giving support all the time as required.*" -P1 |
|  | **1.5 Feasibility of participating in PM+** | | | | |
|  |  | **1.5.1 Accessibility & Affordability** | | Participants mention whether it was feasible for them to attend the sessions when considering the location of the sessions in terms of transportation and money they had to spent | "*In fact, the transportation to the program place was a bit hard for me as there was no bus coming to the front of the municipality so I had to walk for some distance to reach there.*" -P7 |
|  |  | **1.5.2 The timing of the sessions** | | Participants mention whether the timing of the sessions were suitable for them to attend | "*...the timing of the program was very suitable for her*" -R1 |
|  |  | **1.5.3 The impact of daily responsibilities** | | Participants mention whether it was feasible for them to attend the sessions when considering the other responsibilities | "*Yes actually, as I have kids, and leaving them for a couple of hours was hard for them and their father and actually my husband did not totally approve of me going for this program because of the kids.*" -P7 |
|  |  | **1.5.4 Feasibility of attending the sessions** | | Participants mention whether they came across with any difficulties in general while participating | "*No, thank God I did face not any difficulty and I enjoyed coming to the sessions.*" -P1 |
| **2. Experiences of delivering Group PM+** | | | | | |
|  | **2.1 Facilitators of PM+ delivery** | | | | |
|  |  | **2.1.1 The content of gPM+** | | Participants mention the facilitating aspects of the content | "*So, for get going keep doing, the behavioral activation session, I would say that was one of the most applied strategies. My experience was like that. Because they felt the importance of being behaviorally active and engaging. Also sparing time for themselves, trying to engage in things that they used to enjoy before. That was a good one for them.*" -F2 |
|  |  | **2.1.2 The facilitator of gPM+** | | Participants mention the role of the facilitator as a facilitating factor | "*She already has a problem, an issue, and you show a way to solve that problem. You give a tool but at the same time you follow along with her. You ask her “what did you do here, what did you do there?”. I think even the fact that you're following her motivates her.*" -F1 |
|  |  | **2.1.3 The group format** | | Participants mention the benefits of delivering PM+ in a group format | " *The strength of the group was also great. It was also very beneficial for them to get together and experience it once a week. It made us happy too. When they came back happy or with positive things, we felt like "yeah we did something" or "we're doing something and it's working". That was beautiful too.*" -F1 |
|  |  | **2.1.4 The date, time and location of the sessions** | | Participants mention that when and where the sessions are delivered could be a facilitating factor | "*Maybe some flexible timing. Because you know for males, some of them would be working, looking for work, trying here and there you know. Providing them with a proper timing is the best thing that could help them.*" -F2 |
|  |  | **2.1.5 Motivation of the facilitator** | | Participants describe their own motivation while delivering PM+ | "*It made us happy too. When they came back happy or with positive things, we felt like "yeah we did something" or "we're doing something and it's working". That was beautiful too.*" -F1 |
|  | **2.2 Barriers to gPM+ delivery** | | | | |
|  |  | **2.2.1 Participant related factors** | | | |
|  |  |  | **2.2.1.1 Motivation related factors** | Participants mention that the participants may be willing to attend the program and the sessions for different reasons than improving their well-being | "*Of course, there were some difficulties. Some people were not really believing in what we were doing. Yeah, for some people this was not really interesting, or they had their other issues.*" -F2 |
|  |  |  | **2.2.1.2 Mental health literacy related factors** | Participants mention that people who attended the program had low levels of mental health literacy which may have prevented them to comprehend all aspects of the intervention | "*The whole mental health thing is not really heard of in the community. So, you are giving some details about something that people do not have the basics of.*" -F2 |
|  |  |  | **2.2.1.3 Suitability of the intervention to the participants' expectations from the program** | Participants mention the content and the materials of PM+ could be a barrier since some of them preferred to talk and work on other issues | "*But sometimes they insisted on talking about the pain of losing someone who has died. For example, they wanted to talk about unsolvable problems. But we did not say that "it is not for this program, let's not talk about this subject" directly, but trying to do that was obviously a challenge.*" -F1 |
|  |  | **2.2.2 The structure of gPM+** | | Participants mention that how PM+ was structured and programmed may not be enough or may be too brief for participants to implement the skills they learned | "*I think of it as an obstacle, will it be forgotten? Or it's done and it's over, maybe as they suggested if we meet once a month as a reminder... If we can practice what we've learned, would that help them improve? I think it might be, but here's the thing "I did it and it's done". What's the reminder? The lack of reminders is perhaps a hindrance..*" -F1 |
|  |  | **2.2.3 Adherence** | | Participants mention that the people who are attending the sessions not attending or not implementing the skills could be a barrier | "*I cannot say anything negative. The most negative thing for us was that a participant not participating in the group.*" -F1 |
|  |  | **2.2.4 Management of the group** | | Participants mention the difficulties of managing a group including people from different backgrounds who may have different demands | *“He was like “you guys are giving us this intervention, but nothing is changing in our conditions. Change our condition and we would feel better instead of trying to make us feel better in the same condition”. I understand his point of view but there was this narcissist attitude made things difficult. But it was manageable.”-*F2 |
|  |  | **2.2.5 Reactions of the participants' family members and friends** | | Participants mention that the negative reactions of the closed ones could be barrier | "*Maybe some flexible timing. Because you know for males, some of them would be working, looking for work, trying here and there you know. Providing them with a proper timing is the best thing that could help them.*" -F2 |
|  |  | **2.2.6 Contextual factors** | | Participants mention that the factors related with the context that the PM+ participants are living in such as post-migration living difficulties could be a barrier | "*Also, in a state of where he is now as a refugee, fighting with life, trying to find a loaf of bread… I would say that was not a good one. They did not really apply that, and they would not it in the future.*" -F2 |
|  | **2.4 Suggestions for improvement** | | | Participants had suggestions for the improvement of PM+ in terms of its content or delivery | "*I wish they had a video of course. Of course, this would not be possible, because maybe even with the permission of the group…*" -F1 |

| **Theme** | **Code** | **Subcode** | **Description** | **An illustrative quote from the interviews** |
| --- | --- | --- | --- | --- |
| **1. Scaling up gPM+ (suggestions from key informants)** | | | | |
|  | **1.1 Delivery of gPM+ in PHC** | | | |
|  |  | **1.1.1 Feasibility of delivery of gPM+ in PHC** | Participants discuss the general feasibility of integrating PM+ into primary health care settings | " *I think it can be delivered because PM+ is something like first psychological aid, actually. Therefore, I think it can be delivered very well in primary health care services. These primary health care services may include health centers if there is such a unit. It may include social service centers. -*KI1 |
|  |  | **1.1.2 Facilitating factors related to delivery of gPM+ in PHC** | Participants identify factors that could support the successful implementation of PM+ within PHC | " *The support of decision-makers would help. Let's say a pilot study was conducted, and it was only added to some health centers at first.*" -KI3 |
|  |  | **1.1.3 Barriers related to delivery of gPM+ in PHC** | Participants discuss systemic barriers | "*The cumbersome general structure of the public sector can be a barrier because there are some very ingrained approaches. It may be difficult to change the general perception of public personnel, that is, the perception of elder decision-makers*" -KI1 |
|  | **1.2 Integration of lay facilitators into PHC** | | | |
|  |  | **1.2.1 Facilitators** | Participants mention facilitating factors that would enable integration of lay counselors into PHC | "*It may be easier to call it something else rather than psychological support. This strategy can be useful for getting this support from non-professionals, non-psychologists, and not to get the reaction of people in that professional group.*" -KI2 |
|  |  | **1.2.2 Barriers** | Participants describe barriers that could hinder the integration of lay counselors into primary health care (PHC). | "*I think it would be better to train the staff working in those services rather than bringing someone from outside, you know someone who is not working there. Because it might be difficult for a person who is not working there to get integrated into that system..*" -KI4 |
|  | **1.3 Integration of gPM+** | | | |
|  | **1.3.1 Coverage of psychological problems** | | | |
|  |  | **1.3.1.1 Facilitators** | Participants mention the facilitating factors that would enable the integration of gPM+ in terms of ensuring coverage of psychological problems. | *"PM+ can be beneficial if it is presented as a course to help you deal with your problems, not as psychological support, if it is presented as “we refer you here, it takes 5 weeks anyways”, if people who are already prejudiced to receive mental health services are included in this way."* -KI3 |
|  |  | **1.3.2.1 Barriers** | Participants mention the barriers that would hinder the integration of gPM+ in terms of ensuring coverage of psychological problems. | *" Actually, I don't think PM+ will be enough on its own. PM+ will not be sufficient, as it does not offer a solution to the trauma disorders we see in refugees on its own.”* -KI2 |
|  | **1.3.2 Additional services to be integrated** | | Participants discuss other available services into which gPM+ could be integrated | " *This can be delivered not only in primary health care services in terms of reaching people, but also in institutions such as workplaces*.”-KI1 |

# **S4: Consolidated Criteria for Reporting Qualitative Research (COREQ): 32-item Checklist-Based Evaluation**

| Item | Description | Status |
| --- | --- | --- |
| **Domain 1: Research team and reflexivity** | | |
| *Personal characteristics* | | |
| 1. Interviewer/facilitator | Arabic-speaking bilingual researcher team conducted interviews. | Yes |
| 2. Researcher’s credentials | Researchers' credentials included PhD-level and PhD candidate positions; interviewers were university graduates or senior undergraduate students | Yes |
| 3. Occupation | Research team included doctoral researchers, clinical psychologists, professors, graduates and undergraduates in psychology and public health | Yes |
| 4. Gender | Gender of interviewers reported; research team was listed in author list. | Yes |
| 5. Experience and training | Interviewers were trained in qualitative interviewing. | Yes |
| *Relationship with participants* | | |
| 6. Relationship established | Interviews were conducted independently, no prior contact | Yes |
| 7. Participant knowledge of interviewer | Participants didn’t know interviewers | Yes |
| 8. Interviewer characteristics | Interviewers were independent researchers with no prior relationship to participants | Yes |
| **Domain 2: study design** | | |
| *Theoretical framework* | | |
| 9. Methodological orientation | Framework Method (Gale et al., 2013) specified for data analysis | Yes |
| Participant selection | | |
| 10. Sampling | Purposive sampling described. | Yes |
| 11. Method of approach | Interviews via phone and video stated. | Yes |
| 12. Sample size | 23 participants reported. | Yes |
| 13. Non-participation | No participant refused. | Yes |
| *Setting* | | |
| 14. Setting of data collection | Setting (phone/video) described. | Yes |
| 15. Presence of non-participants | Interviews conducted privately. | Yes |
| 16. Description of sample | Key characteristics of the sample reported. | Yes |
| *Data collection* | | |
| 17. Interview guide | Topic guide included as supplement. | Yes |
| 18. Repeat interviews | No repeat interviews were conducted. | Yes |
| 19. Audio/visual recording | Not recorded due to legal restrictions; explained. | Yes |
| 20. Field notes | Interviewers took field notes. | Yes |
| 21. Duration | Interviews lasted 45–60 minutes. | Yes |
| 22. Data saturation | Data saturation was reached. | Yes |
| 23. Transcripts returned | Member checking not done; acknowledged as limitation. | No |
| **Domain 3: analysis and findings** | | |
| *Data analysis* | | |
| 24. Number of data coders | Two coders involved. | Yes |
| 25. Description of coding tree | Thematic categories described. | Yes |
| 26. Derivation of themes | Both inductive and deductive approaches explained. | Yes |
| 27. Software | NVivo 11 used. | Yes |
| 28. Participant checking | Member checking not done; acknowledged as limitation. | No |
| *Reporting* | | |
| 29. Quotations presented | Quotes were presented to illustrate the themes. | Yes |
| 30. Data and findings consistent | Results supported by quotes (data). | Yes |
| 31. Clarity of major themes | Major themes clearly outlined. | Yes |
| 32. Clarity of minor themes | Minor themes and dissenting views discussed. | Yes |
